# Provision of comfort care amidst quicksand: A scoping review of the common elements in neonatal end-of-life care

**DOI:** 10.34172/hpp.025.43062

**Published:** 2025-12-30

**Authors:** Aysha Jawed, Michelle Mowry, Catherine Ehrhardt

**Affiliations:** ^1^Department of Pediatrics, Johns Hopkins University School of Medicine, Baltimore, Maryland, USA; ^2^Center for Excellence in Public Health Leadership, Kennedy Krieger Institute, Baltimore, Maryland, USA; ^3^Department of Pediatric Nursing, Johns Hopkins Children’s Center, Baltimore, Maryland, USA

**Keywords:** Bereavement, Infant mortality, Pain management, Palliative care, Quality-of-life, Terminal care

## Abstract

**Background:**

Neonatal end-of-life (EOL) care remains a complex challenge in modern healthcare. Increasing numbers of infants with life-limiting illnesses are dying at home or in hospitals, with or without hospice involvement. Despite numerous reviews, commentaries, case reports, and original studies addressing neonatal EOL care, there are currently no standardized guidelines. This gap complicates the quality and delivery of care during this fragile phase.

**Methods:**

We conducted a comprehensive review of published literature on neonatal EOL care, including systematic reviews, observational studies, and expert recommendations. Common elements were identified across these sources to inform future research directions. Our analysis focused on recurring themes related to symptom management, family-centered care, ethical considerations, and interdisciplinary collaboration.

**Results:**

The literature consistently highlights key components of neonatal EOL care: effective pain and symptom control, clear communication with families, psychosocial support, and involvement of multidisciplinary teams. However, significant variability exists in implementation across settings. Few studies provide longitudinal or prospective data, and most recommendations lack empirical validation, underscoring the need for standardized approaches.

**Conclusion:**

Current evidence suggests that harmonizing neonatal EOL practices could improve care quality and reduce fragmentation in EOL care coordination. We propose feasibility studies and prospective research to evaluate these common elements for integration into evidence-based guidelines. Such efforts align with the World Health Organization’s (WHO’s) mission to enhance EOL care globally, ensuring dignity and comfort for neonates and their families.

## Introduction

 Infant mortality is a significant global health issue, with half of all pediatric deaths occurring during infancy and half of those in neonates.^1^ The majority of infant deaths are occurring in hospitals, without hospice engagement.^1^

 Many infants with life-limiting illness at the end-of-life (EOL) die in the neonatal intensive care unit (NICU).^2^ In one study, 61% of child deaths occurred in infants less than one year old, with nearly 30% dying in the NICU.^3^ In one study among 56 parents of children who had died in an intensive care unit (ICU) setting, 55% of them felt that they had either limited or no control during their child’s final days of life.^4^ Furthermore, most parents in this study felt that they were not able to participate in caring for their child at the EOL amidst the ICU conditions.^5^

 There is significant variability in neonatal EOL guidelines and quality assessment. For example, several systematic reviews and original research studies have presented mixed findings on initiation of specific medications and dosing based on clinical status of children at the EOL.^6-13^ There have been different interpretations of presenting symptoms (e.g. related to neuroirritability) which makes it more difficult to provide pain and symptom management.^8,9,11,14,15,16-19^ There have also been different parameters for initiating palliative sedation and compassionate extubation among children at the EOL.^7,9,11,20,21^ These inconsistencies in findings have contributed to the challenges in strengthening the quality of life for the children at the EOL. Furthermore, these inconsistencies also heighten the risk of the quality and delivery of care along with potentially contributing to disparities in the care of these children based on illness and sociodemographic characteristics.

 Most US healthcare systems lack neonatal EOL guidelines for optimizing symptom control, pain relief, and quality of life. Among the scant hospitals which have implemented guidelines, there is substantial variation in practice which contributes to the gap in knowledge, research, and practice. Consequently, there are no standardized neonatal EOL guidelines in existence to date in the United States, and there are extensive variations in practice across healthcare institutions and hospice programs with their own guidelines which have further exacerbated informing care for these patients.

 Advances in medicine have increased survival for children with complex medical needs, highlighting the importance of optimizing EOL care, including pain and symptom management, psychosocial support, and family-centered care. In addition, transitioning infants from the intensive care setting to the general pediatric ward reduces high-cost healthcare utilization, yielding value to the healthcare system and increased comfortability for the family. Through conducting an extensive review of the existing literature on varied neonatal EOL recommendations implemented across different healthcare systems, we have identified the common elements and have synthesized them as targets for intervention in informing future research and practice to create a more harmonized and standardized approach in neonatal EOL care.

## Methods

###  Search Strategy

 A comprehensive literature review was conducted in March 2025 to identify studies on neonatal EOL care. The following academic databases were reviewed: EBSCO, ERIC, Academic Search Ultimate, PubMed, Medline, APA PsychInfo. CINAHL, Embase, Scopus, Google Scholar, and Cochrane Review. Key words and descriptors that formed the search strategy to uncover articles for this review were “neonatal palliative care”, “pediatric palliative care”, “infant mortality”, “infant morbidity”, and “neonatal hospice”.

###  Eligibility Criteria 

 Sources integrated in this review included content specifically pertaining to guidelines, measures, and protocols in the care of infants at the EOL on a continuum across hospital and community contexts. Any articles that did not include neonatal EOL care considerations across these contexts were excluded from this review.

###  Procedure

 Three authors independently screened titles and abstracts of the retrieved articles. Any differences pertaining full-text inclusion were resolved through consensus among the research team. Next, the authors independently abstracted data across all included sources on guidelines, measures, and protocols in the care of infants at the EOL that also accounted for any additional descriptive and qualitative information on the nature and implementation of these interventions and their limitations. Data were collected using a Standardized Data Extraction Form (SDEF) developed for this review. The form captured author(s), publication year, study design, sample size, setting, key findings, and reported limitations for each included study. Using the SDEF ensured consistency across reviewers, minimized bias, and enhanced the reliability and rigor of the synthesis process. Findings, trends, developments, and themes were subsequently compared and discrepancies were resolved through active discussions amongst the authors.

 The methodological quality of the included studies was evaluated using standardized appraisal tools. For qualitative studies, the Critical Appraisal Skills Programme (CASP) checklists were applied to assess aspects such as clarity of aims, appropriateness of design, and rigor of data analysis. For quantitative studies, the Cochrane Collaboration’s Risk of Bias tool was used to examine potential biases in randomization, blinding, and outcome reporting. These tools ensured a systematic and transparent approach to evaluating study validity and reliability.

## Results

**Figure 1:**
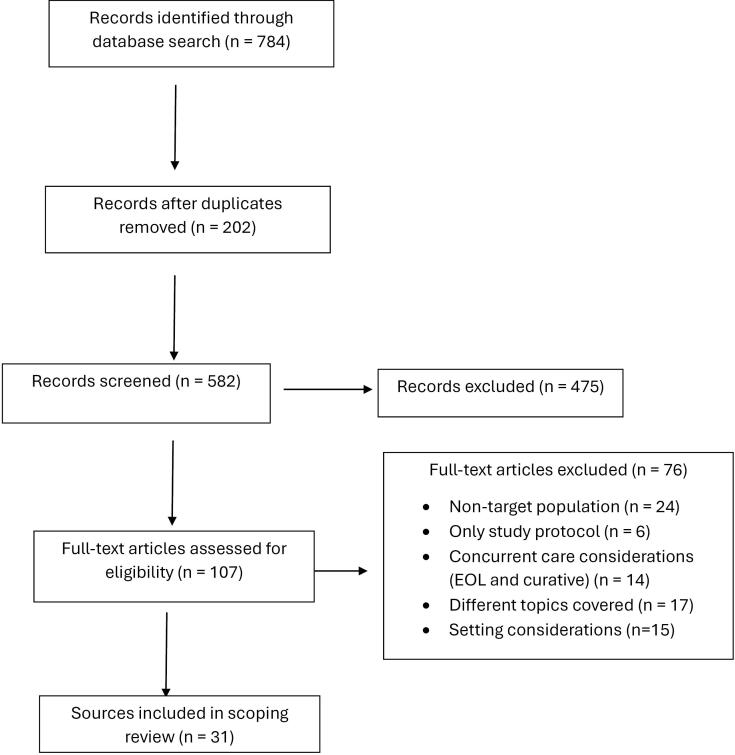
Literature review flowchart

 A cumulative total of 784 records were identified across the databases reviewed from the past 30 years. 202 of these records were duplicates and ultimately excluded. Among the remaining 582 records, 475 of them were subsequently excluded for one or more of the following reasons: (1) did not contain full-text articles; (2) intervention components did not involve delivery of EOL care to infants, and (3) accounted for older pediatric populations. 107 remaining full-text articles were examined for inclusion in this scoping review. 76 of them were ultimately further excluded for the following reasons: (1) nontarget population; (2) presented only a study protocol; (3) presented concurrent care considerations (EOL and curative), (4) involved perceptions of healthcare providers rather than utilization of comfort care measures, and (5) did not involve EOL care considerations on a continuum across hospital and community contexts. 31 of them ultimately met the criteria for presenting guidelines, protocols and measures on neonatal EOL care as elucidated in Figure 1. A consolidated breakdown of each of these source’s elements in the delivery of EOL care along with the measures to assess each intervention can be found in Table 1.

**Table 1:** Common elements for the standardization of neonatal end-of-life guidelines

**Content area**	**Recommendations**	**Measure**
Medication administration	Opiates^6-13^; Anticholinergics^6,9,12^; Anticonvulsants^6,9,12^; Antipyretics^6,9,12^; Benzodiazepines^6,9,12^; Diuretics^6,9,12^; Hypnotics^6,9,12^; Occasional use of (glycopyrronium or hyoscine)^7^; Oral or nasogastric morphine^7^; Oral sucrose (24%) as per pain protocol^7,9^; Paracetamol^7,11^; Sedatives^8-10,13^; Medications to relieve air hunger^9^; Morphine^9^; Paralytics^9^; Alternative medications^11^; Anxiolytics^11^; Higher doses may be needed to achieve symptom management^11^; Narcotic and non-narcotic analgesics^11^; Gabapentin^22-25^	Aggregated clinical and administrative data; Audits; Case reviews; Clinician reports; Descriptive observational data from clinical and quality assessments; Goal concordance (kappas); Health record reviews; Pain and comfort scores; Patient and caregiver reports; Reliability and validity scores; Scoring system for quality indicator of service
Clinical documentation	Symptoms, severity of symptoms, indications for interventions, interventions implemented to alleviate symptoms, reassessment of symptoms post-intervention^11,26^	Aggregated clinical and administrative data; Audits; Case reviews; Clinician reports; Descriptive observational data from clinical and quality assessments; Goal concordance (kappas); Health record reviews; Pain and comfort scores; Reliability and validity scores; Scoring system for quality indicator of service
Palliative Sedation	Anxiolysis^11^; Deep sedation with loss of consciousness^11^; High symptom burden^11^; In palliative sedation to the unconscious, agents commonly used included propofol, phenobarbital, ketamine, and dexmedetomidine. A hypnotic is often included after all measures to treat symptoms have been exhausted and is administered along with the current regimen^11^; Medication titration in palliative sedation - Many providers use the following guide to make medication increases that are proportional to the severity: mild breakthrough - 10 to 20% increase, moderate breakthrough – 20 to 30% increase, and severe – 30 to 50% increase^11^; Palliative sedation to the unconscious - the intent is to induce loss of consciousness to eliminate suffering^11^; Traditional treatment options have not achieved control of symptoms^11^; Decrease and control symptom burden^20,21^; Has been referred to as terminal sedation, palliative sedation therapy, controlled sedation for intractable patients, EOL sedation, and continuous sedation for the dying^20,21^	Aggregated clinical and administrative data; Audits; Case reviews; Clinician reports; Descriptive observational data from clinical and quality assessments; Goal concordance (kappas); Health record reviews; Pain and comfort scores; Patient and caregiver reports; Reliability and validity scores; Scoring system for quality indicator of service
Non-Pharmacological Measures	Breastfeeding^7^; Gentle suctioning^7,9,12,27,28^; Swaddling^7,30^; Skin care^9,12,27,28^; Body thermal stability^11^; Involves holding^11^; Without medications^11^; Promotes Skin-to-skin contact^11-12,16,27,28^; Decreasing stimulation^11,12,27,28^; Repositioning^11,12,27,28^; Promotes bonding^11,29^; Elevating the head^12,27,28^; Fluid restriction^12,27,28^; Massage^12,27,28^; Mouth care^12,27,28^; Non-nutritive sucking^30^; Nonspecific^31^	Aggregated clinical and administrative data; Audits; Case reviews; Clinician reports; Descriptive observational data from clinical and quality assessments; Goal concordance (kappas); Health record reviews; Pain and comfort scores; Patient and caregiver reports; Reliability and validity scores; Scoring system for quality indicator of service
Symptoms	Being unsettled and agitated^7^; Furrowing of the brow and squeezing shut of eyes^7^; Persistent crying^7^; Tachycardia^7^; Seizures^7,11^; Agitation - autonomic signs, increased motor activity, restlessness, and disturbed or disrupted sleep^8,9,11,14-19^; Neuroirritability - autonomic signs, increased motor activity, restlessness, and disturbed or disrupted sleep^8,11,14-15,17-19^; Dyspnea - discomfort with breathing^8,11,14-19^; Increased secretions^8,11,17^; Pain - could be chronic, subacute, nociceptive pain (tissue damage or inflammation which can be somatic, localized to a specific region, or visceral which affects the internal organ), neuropathic pain (damage or irritation to the nerve)^8,11,17^; Discomfort^9^; Reliant on a subjective report either by the patient or a visual assessment by another individual based on markers such as respiratory rate, the presence of hypoxia, and work of breathing^9^; Shortness of breath, such as nasal flaring, air hunger, color changes, or grunting^9^; Abnormal movements^11^; Disturbed sleep^11^; Restlessness^11^	Aggregated clinical and administrative data; Audits; Case reviews; Clinician reports; Descriptive observational data from clinical and quality assessments; Goal concordance (kappas); Health record reviews; Pain and comfort scores; Patient and caregiver reports; Reliability and validity scores; Scoring system for quality indicator of service
Durable medical equipment	Parents may wish to administer oxygen as a comfort measure^9^ Suction machine or bulb suction^9^	Aggregated clinical and administrative data; Audits; Case reviews; Descriptive observational data from clinical and quality assessments; Goal concordance (kappas); Health record reviews; Pain and comfort scores; Patient and caregiver reports; Reliability and validity scores; Scoring system for quality indicator of service
Spatial Considerations	The best available physical space with privacy and comfort should be chosen^9^; Whether the infant is moved to a room off the unit (e.g., a family room), onto a general pediatrics ward, or kept on the postpartum floor^9^	Aggregated clinical and administrative data; Audits; Reliability and validity scores; Pain and comfort scores; Patient and caregiver reports; Descriptive observational data from clinical and quality assessments; Case reviews; Goal concordance (kappas); Health record reviews; Scoring system for quality indicator of service
Medical De-Escalation	Agreement to cease all invasive care, including cessation of frequent vital signs, monitoring, medical machinery, and artificial feeding^9^; Supplemental oxygen is usually not given when a ventilator is withdrawn^9^; Decreasing painful procedures^11,28^; Do Not Resuscitate Orders^31^	Aggregated clinical and administrative data; Audits; Case reviews; Clinician reports; Descriptive observational data from clinical and quality assessments; Goal concordance (kappas); Health record reviews; Pain and comfort scores; Patient and caregiver reports; Reliability and validity scores; Scoring system for quality indicator of service
Access	IV access as continuous or intermittent infusions^6,9^; Buccal^7,9^; Subcutaneous^7,9^; Oral administration^7,11^; Suppository^9^; Intranasal^11^; Transdermal^11^	Aggregated clinical and administrative data; Audits; Case reviews; Clinician reports; Descriptive observational data from clinical and quality assessments; Goal concordance (kappas); Health record reviews; Pain and comfort scores; Patient and caregiver reports; Reliability and validity scores; Scoring system for quality indicator of service
Education for clinicians	Adapting and tailoring care^32^; Clinical knowledge^32^; Leadership^32^; Medical support^32^; Morals, values, and beliefs^32^	Aggregated clinical and administrative data; Audits; Case reviews; Clinician reports; Descriptive observational data from clinical and quality assessments; Goal concordance (kappas); Health record reviews; Reliability and validity scores; Scoring system for quality indicator of service
Bereavement support	Care after death of the baby^7^; Attunement: attunement to the family’s needs could create opportunities for families to bond with their infants^32^; Bonding: understanding the needs of the infant and families allow nurses to provide a safe environment for bonding and memory-making^32^; Communication to parents - providing clear information to families throughout the palliative care period, providing education to parents to support the decision-making process^32^; Gift of time^32^; Personalized care – in line with family preferences^32^; Post-death information^32^; Supporting the extended family: immediate emotional support of the family, but also the long-term impact of this support on the family into the future^32^	Aggregated clinical and administrative data; Audits; Case reviews; Descriptive observational data from clinical and quality assessments; Goal concordance (kappas); Health record reviews; Pain and comfort scores; Patient and caregiver reports; Reliability and validity scores; Scoring system for quality indicator of service
Patient-and-family-centered care	Goals of care for feeds and resuscitation related to acute deterioration, choice of place of care in the event of illness, care if the infant dies at home, death certification and post-mortem investigations (if appropriate)^7^; Multidisciplinary discharge planning meeting is arranged between the professionals (community palliative care team, continuing care team, neonatal outreach, hospice care team, among others)^7^; Visitation - At this time parents may want to have their friends and family around them, visiting will be unrestricted during this time and we should ensure they have space and privacy to grieve^7^; Communication^7,32^; Facilitating prenatal end-of-life discussions for families who learn about a life-limiting diagnosis for future infant^9^; Hospital social worker involvement^9^; Individual preferences^9^; Tangible assistance - meal tickets, assistance with parking fees, a phone card to allow families to call their extended families, access to transportation, a place to stay (preferably with the infant), financial assistance as part of their overall care, day care for the other siblings, any other practical support issues that can be met should be met, and those that cannot be met should be explained^9^; Emotional support^32^	Aggregated clinical and administrative data; Audits; Case reviews; Descriptive observational data from clinical and quality assessments; Goal concordance (kappas); Health record reviews; Pain and comfort scores; Patient and caregiver reports; Reliability and validity scores; Scoring system for quality indicator of service
Preferred place for end-of-life care	Hospital, the hospice or home^7^	Aggregated clinical and administrative data; Audits; Case reviews; Clinician reports; Descriptive observational data from clinical and quality assessments; Goal concordance (kappas); Health record reviews; Pain and comfort scores; Patient and caregiver reports; Reliability and validity scores; Scoring system for quality indicator of service
Religious aspects for spiritual support	Alternatively, families own religious representatives can visit the unit to provide spiritual support for the family and religious ceremonies or prayers can be facilitated^7^; It is best to ask the family about their customs and beliefs^7^; Sensitivity to these issues will bring comfort and support to those who need it^7^; Trust multi-faith representatives are accessible^7^; Spiritual welfare^31^	Aggregated clinical and administrative data; Audits; Case reviews; Clinician reports; Descriptive observational data from clinical and quality assessments; Goal concordance (kappas); Health record reviews; Pain and comfort scores; Patient and caregiver reports; Reliability and validity scores; Scoring system for quality indicator of service
Nursing care orders	Nonspecific^31^	Aggregated clinical and administrative data; Audits; Case reviews; Clinician reports; Descriptive observational data from clinical and quality assessments; Goal concordance (kappas); Health record reviews; Pain and comfort scores; Reliability and validity scores; Scoring system for quality indicator of service
Feeding and nutrition	Enteral or parenteral medications^7^; Intravenous nutrition and hydration can safely be discontinued in any infant on an end-of-life care pathway after agreement with parents and other professionals^7^; Oral nutrition should only be withheld if it is felt that providing it will cause pain or discomfort. The amount of feeds should be determined by their clinical condition. In some situations, it may be appropriate to allow the infant to suckle at the breast (if they are able to do so). If the mother / parents are keen to offer breast milk to the neonate, they should be supported through expression of breast milk. In infants who are expected to die soon, it may be appropriate to stop all enteral feeds^7^; Participation in discussion on the appropriateness of feeding, and prevention of air hunger^9^; Nonspecific^31^	Aggregated clinical and administrative data; Audits; Case reviews; Clinician reports; Descriptive observational data from clinical and quality assessments; Goal concordance (kappas); Health record reviews; Pain and comfort scores; Patient and caregiver reports; Reliability and validity scores; Scoring system for quality indicator of service
Compassionate extubation	After extubation, parents should be allowed time in the rooms with their baby^7^; Air and oxygen points to help provide some time on a ventilator or other breathing support^7^; The baby should be assessed regularly for pain and distress and adequate analgesia/ sedation should be provided^7^; Important to talk about potential responses from the baby, uncertainties about duration of survival after extubation, nutrition and analgesia following extubation^7^; Parents should be allowed time before they feel ready for the extubation and should be supported by the multidisciplinary team throughout the process^7^; Affirming this with their spiritual leaders and extended family members is also important, as this is a difficult concept for some families to comprehend and may take recurrent discussion^9^; If the transition in care involves the removal of ventilatory support, explain that the use of ventilators is for the improvement of heart –lung conditions until cure — when cure is a likely outcome^9^; Make clear that using a ventilator to breathe for an infant who is overwhelmed by the underlying disease process, and is dying, is neither beneficial nor recommended^9^; Parents can hold a service at the bedside as desired^9^; Staff, including the social worker, neonatologist, spiritual advisor, and primary nurse, should be close by and available upon request^9^	Aggregated clinical and administrative data; Audits; Case reviews; Clinician reports; Descriptive observational data from clinical and quality assessments; Goal concordance (kappas); Health record reviews; Pain and comfort scores; Patient and caregiver reports; Reliability and validity scores; Scoring system for quality indicator of service

 Through reviewing these sources, common elements included the following content categories: 1) dosing and administration of pain medications (e.g. opiates, morphine and other narcotics, sedatives, benzodiazepines, gabapentin, diuretics, anticonvulsants, antipyretics); 2) clinical documentation on pain and symptom management; 3) parameters for palliative sedation (e.g. medication titration for levels of palliative sedation); 4) nonpharmacological measures (e.g. skin-to-skin contact, bonding, mouth and skin care, repositioning, limiting stimulation, gentle suctioning, massaging); 5) clinical assessment of symptoms (e.g. autonomic signs, increased motor activity, restlessness, and disturbed or disrupted sleep for agitation and neuroirritability, discomfort with breathing for dyspnea, nasal flaring, air hunger, color changes, or grunting for shortness of breath, pain classifications); 6) utilization of durable medical equipment; 7) spatial considerations for EOL care; 8) medical de-escalation recommendations (e.g. discontinuation of all invasive care related to feeding, taking vitals, and medical machinery, utilization of DNR orders); 9) forms and parameters of access (e.g. intravenous access for continuous or intermittent infusions, oral, subcutaneous); 10) education for clinicians; 11) bereavement support (e.g. bereavement cart); 12) patient-and-family-centered care; 13) preferred place for EOL care; 14) religious aspects for spiritual support; 15) nursing care orders; 16) feeding and nutrition (e.g. discontinuation of intravenous nutrition and hydration, cease oral feeding if creating pain and discomfort for infant); and 17) compassionate extubation (e.g. assuring psychosocial support for family before, during and after, quality of life considerations for extubation).

 Across many of these sources, each quality indicator was either measured or recommended to be measured by one or more of the following: aggregated clinical and administrative data, reliability and validity scores, pain and comfort scores, patient and caregiver reports, audits, clinician reports, descriptive observational data from clinical and quality assessments, case reviews, health record reviews, scoring system for quality indicator of service, and goal concordance (kappas). Table 1 presents a comprehensive breakdown of each of these content categories for the common elements. Figure 1 delineates the process of determination for inclusion of articles in this review.

###  Medication Administration

 There was a wide range of medications covered for administration and consideration for infants at the EOL. Opiates were recommended in EOL care for infants in eight sources.^6-13^ Sedatives were covered for administration in four sources.^8-10,13^ Benzodiazepines, diuretics, hypnotics, anticonvulsants, anticholinergics, and antipyretics were supported by three sources.^6,9,12^ Gabapentin was accounted for across four sources.^22-25^ Oral sucrose (24%) as part of a pain protocol was recommended in two sources.^7,9^ In addition, there were two sources that reviewed utilization of paracetamol.^7,11^ Alternative medications with no specific names indicated were addressed in one source.^11^ This specific source also suggested that higher doses could be needed to achieve symptom management. Morphine, paralytics, medications to relieve air hunger, narcotic and non-narcotic analgesics, and anxiolytics were covered in another source.^9^ Glycopyrronium or hyoscine on an occasional basis was reviewed in one source.^7^ The same source specified the morphine was provided to infants via oral or nasogastric administration.^7^

###  Clinical Documentation

 Clinical documentation was clearly described in two sources and specifically noted symptoms, severity of symptoms, indicators for interventions, interventions implemented to alleviate symptoms, and reassessment of symptoms post-intervention.^11,26^

###  Palliative Sedation

 Palliative sedation was covered in multiple sources. In one source, palliative sedation was reviewed as a measure to alleviate high symptom burden and further recommended for utilization when traditional treatment options were not effective in achieving symptom control. In addition, palliative sedation in this source also encompassed deep sedation with loss of consciousness and anxiolysis.^11^ Two additional sources also recommended palliative sedation in decreasing and controlling symptom burden either as a terminal form, controlled for intractable patients, or continuously at the EOL.^20,21^

 In one source, medication titration was also examined as an integral part of palliative sedation. There were thresholds to guide medication increases proportionate to severity of pain (for e.g. 10-20% increase with mild breakthrough, 20-30% increase with moderate breakthrough, and 30-50% increase with severe breakthrough in this source).^11^ In addition, integrating palliative sedation to the unconscious for inducing loss of consciousness as the basis to eliminate suffering was also reviewed.^11^ Examples of medications recommended for the unconscious were propofol, phenobarbital, ketamine, and dexmedetomidine.^11^ If none of these medications were successful in symptom control across instances, then hypnotics were supported as the last line of sedation alongside the current regimen.^11^

###  Non-Pharmacological Measures 

 Non-pharmacological measures were also covered in several sources. Five sources integrated content on skin-to-skin contact.^11,12,16,27,28^ In addition, skin care was supported in four sources.^9,12,27,28^ Gentle suctioning was also recommended in five sources.^7,9,12,27,28^ Repositioning and decreasing stimulation were addressed in four sources.^11-12,27,28^ Three sources reviewed fluid restriction, elevating the infant’s head, massages, and mouth care.^12,27,28^ Two sources supported measures to promote bonding.^11,29^ Content on swaddling was integrated in two sources.^7,30^ Achieving body thermal stability, holding infant, and taking measures to reduce consideration of medication were covered in one source.^11^ Non-nutritive sucking was also addressed in one source.^30^ Another source promoted breastfeeding.^7^ Lastly, one source recommended nonpharmacological measures but was nonspecific about which ones.^31^

###  Symptomology 

 Multiple symptoms were accounted for across many sources. Agitation as manifested by autonomic signs, increased motor activity, restlessness, and disturbed or disrupted sleep was covered in nine sources.^8,9,11,14-19^ In addition, specifically neuroirritability in the form autonomic signs, increased motor activity, restlessness, and disturbed or disrupted sleep was addressed in seven of these sources.^8,11,14,15,17-19^ Content on dyspnea was reviewed in eight sources.^8,11,14-19^ Pain manifestation was examined in three sources and was classified as either chronic, subacute, nociceptive (tissue damage or inflammation which can be somatic, localized to a specific region, or visceral which affects the internal organ) pain, or neuropathic pain (damage or irritation to the nerve).^8,11,17^ Increased secretions were also accounted for in these sources.^8,11,17^ Seizures were covered in two sources.^7,9^ Persistent crying, furrowing of the brow, squeezing shut of eyes, being unsettled and agitated, and tachycardia were also examined in one source.^7^ Abnormal movements and disturbed sleep were addressed in one source.^11^ Shortness of breath in the form of nasal flaring, air hunger, color changes or grunting were covered in one source.^9^ Respiratory distress (including hypoxia and work of breathing) was noted in the same source.^9^ Discomfort was also accounted for in this source.^9^

###  Utilization of Durable Medical Equipment 

 Durable medical equipment was addressed in one source. Specifically, this source supported utilization of oxygen, suction machine or bulb suction as comfort measures for infants at the EOL.^9^

###  Spatial Considerations 

 Spatial considerations were reviewed in one source. Specifically, this source addressed whether an infant is moved to a room off the inpatient unit, for example onto a general pediatric ward or continues to remain hospitalized on a postpartum unit.^9^ In addition, the same source also accounted for assuring that the optimal physical space with increased privacy and comfort could be accessible for families.^9^

###  Medical de-Escalation

 Medical de-escalation was addressed in four sources. Two sources accounted for decreasing painful procedures.^11,28^ One source recommended limited provision of supplemental oxygen as an intervention in instances when ventilator support would be withdrawn.^9^ In the same source, content on medical de-escalation also covered cessation of all invasive care (e.g. discontinuation of clinical assessments in the form of frequent vital signs, monitoring, medical machinery, and artificial feeding).^9^ In another source, utilization of the Do Not Resuscitate Order was integrated into EOL care.^31^

###  Methods of Access

 Methods of access were covered in three sources. Two sources reviewed acquisition of IV access for both intermittent and continuous infusions.^6,9^ Oral administration was addressed in two sources.^7,11^ Buccal and subcutaneous access were recommended in two sources.^7,9^ One source accounted for intranasal and transdermal access.^11^ Lastly, another source supported the utilization of suppository for access.^9^

###  Education for Clinicians

 Education for clinicians was accounted for in one source. Specifically, this source covered content on leadership, clinical knowledge, morals, values, and beliefs, adapting and tailoring care, and medical support.^32^

###  Bereavement Support 

 Bereavement support was addressed in two sources. In one source, attunement to the family’s needs was a focus as the basis to create opportunities that promote bonding between family and infant.^32^ Bonding was also subsequently recommended as a facilitator for nurses to provide a safe secure environment for families to engage in memory-making as part of optimizing the gift of time among families with their infants.^32^ Support for extended family as a predictor of support to the immediate family in the future was also a direction of bereavement support covered in this source.^32^ Clear and consistent communication to support caregivers navigate decision-making for their infants at the EOL was also an integral part of bereavement support addressed in this source.^32^ In addition, personalized care that was in line with family preferences was also a focus of bereavement support in the same source.^32^ Provision of post-death information was another form of bereavement support accounted for in this source.^32^ In another source, care after death of the baby was reviewed as a significant part of bereavement support.^7^

###  Patient-and-Family-Centered Care

 Patient-and-family-centered care was covered in three sources. Unrestricted visitation with the infant at the EOL was accounted for in one source with provisions for space and privacy recommended for grieving.^7^ This source also supported multidisciplinary discharge planning meetings among community and inpatient care providers.^7^ In addition, this source also proposed that during these care plan meetings and throughout the EOL phase for infants, goals of care could be consistently reviewed pertaining to feeding, resuscitation attributed to acute deterioration, place of care, EOL care at home, and death certification and post-mortem investigation discussions.^7^ In another source, provision of tangible assistance to families was recommended that specifically addressed their psychosocial needs, including financial support, meal tickets, parking assistance, phone card coverage, access to transportation, lodging, child care and other practice support to meet daily needs of families.^9^ This source also accounted for individual preferences of families, prenatal discussions for families who learned about life-limiting diagnosis of infant prior to birth, and involvement of hospital social worker as part of the provision of patient-and-family-centered care. One source covered emotional support for families^32^. Lastly, two sources accounted for supportive communication with families.^7,32^

###  Preferred Place for end-of-Life Care

 One source accounted for preferred place for EOL care. This source specifically addressed assessment on whether families preferred for their infant to die in the hospital, inpatient hospice, or home.^7^

###  Spiritual Support 

 Spiritual support was covered in two sources.^7,31^ One source reviewed the accessibility of trusted multi-faith representatives for families as well as assurance that religious representatives selected by families could alternatively visit to provide spiritual support for family and infant that include religious ceremonies or prayers.^7^ This source also supported assessments of customs and beliefs among families of infants as the basis to provide culturally sensitive care that is comforting and supportive to families during this vulnerable time.^7^

###  Nursing Care Orders 

 Nursing care orders reviewed were nonspecific in one source.^31^

###  Feeding and Nutrition

 Feeding and nutrition were covered in three sources.^7,9,31^ Enteral or parenteral medications were integrated in one source.^7^ Withholding of oral nutrition was accounted for as a measure in this source if feeding could create any pain or discomfort for infant. In addition, this source supported determination of quantity of feeds based on the clinical condition of the infant. Breastfeeding was also recommended in the same source. This source also supported cessation of enteral feeds for infants expected to die imminently.^7^ Lastly, discontinuation of intravenous nutrition and hydration for infant at the EOL were also covered in this source stemming from goals of care discussions between families and healthcare providers.^7^ Similarly, another source addressed goals of care discussions on feeding in addition to prevention of air hunger.^9^ A different source also referenced feeding and nutrition but covered content that was nonspecific.^31^

###  Compassionate Extubation

 Compassionate extubation was explored in two sources. In these two sources, time for family to prepare and be surrounded with support including from their extended family members, multidisciplinary team, and spiritual leaders was identified as a crucial determinant pre and post extubation.^7,9^ Ensuring that families had the capacity to hold a memorial service for infant at bedside was also accounted for in one source.^9^ Support further recommended in both of these sources included anticipatory guidance for families about possible responses from infant following extubation (e.g. uncertainty about duration of survival, nutrition and analgesia).^7,9^ In one source, air and oxygen points for ventilator and breathing support during this process were also addressed.^7^ In a different source, communication on the futility of a ventilator to support breathing for a dying infant was also covered.^9^ Time with infant following extubation was also an integral factor reviewed in one source.^7^ In addition, assessing the baby consistently during and after extubation for pain and distress was accounted for as the basis to assure appropriate analgesia and sedation for infants in this source.^7^

## Discussion

 Based on these common elements, we propose recommendations to continue work in this domain. Specifically, our recommendations pertain to developing and conducting feasibility, longitudinal, and prospective studies to assess the efficacy of each common element including pain and symptom management, improving quality of life at the end of life, and pharmacologic and nonpharmacologic strategies. Findings across future studies could further uncover consistency, validity, and reliability across one or more of these common elements as the basis for consideration of integration into the development of a constellation of evidence-based guidelines for neonates at the EOL. Feasibility studies could further involve implementing developed guidelines in practice for infants at the EOL engaged in care across healthcare systems with current hospice involvement or eligibility for hospice.

 Another direction from continued research could involve assessing adherence with guidelines among deliverers of care to these infants in a range of ways that include observational studies, audits, and national surveys as the basis to increase the uptake of knowledge and practice in improving quality of life for infants at the EOL. Adherence to evidence-based guidelines improves care outcomes by ensuring consistent, safe, and effective practices, thereby leading to better symptom control, fewer unnecessary interventions, and enhanced family support. Measurable indicators include the proportion of infants with documented EOL care plans, timely pain assessments, and family meeting documentation, as well as outcome measures such as reduced invasive procedures and improved comfort scores. By taking a common elements based approach through identifying each element as a quality indicator, it is possible to further explore directions to create a gold standard in the care of these infants and in turn address a longstanding gap in the clinical practice across the neonatal EOL population.

 While the common elements offer a broad scope for evidence-based neonatal EOL care, guidelines must remain adaptable to the diverse needs of this fragile population. Furthermore, accounting for common elements in EOL care across diverse racial and ethnic groups can help reduce disparities by promoting equity in quality and delivery of care. This can be achieved through culturally adapted standardized care pathways guided by frameworks such as the National CLAS Standards, alongside equity-focused quality improvement models that embed disparity monitoring into routine practice. Incorporating family-centered shared decision-making, supported by linguistically appropriate tools, ensures inclusive engagement. Data-driven approaches, including equity dashboards and predictive analytics, enable proactive identification of gaps, while partnerships with community organizations foster culturally sensitive practices. Finally, embedding cultural humility and bias mitigation training within clinical education, coupled with policy alignment to ethical principles of distributive justice, strengthens systemic efforts to ensure equitable EOL care for all infants.

 Development of a model of community care to increase equitable access to neonatal EOL care could yield promise in streamlining a process for both community hospice and healthcare systems in supporting infants at the EOL across both contexts. Community care in neonatal EOL involves a coordinated approach that bridges healthcare systems and community hospice to ensure equitable, family-centered support. Healthcare systems provide specialized clinical oversight, care planning, and resources, while community hospice delivers in-home comfort care, counseling, and continuity beyond the hospital. Collectively, they streamline transitions, reduce fragmentation, and empower families with compassionate, culturally sensitive choices. It follows that future work in this domain could continue to heighten assurance in the quality and delivery of care for all infants at the EOL through increased collaboration with community hospice and inpatient contexts on a continuum which also further contributes towards the larger goals of the World Health Organization (WHO) in creating a stronger and more competent global palliative care workforce.^33^

 In addition, accounting for these elements could also further support exploration of whether any of them may already be in existence across different academic and community healthcare systems nationally and globally as the basis to inform standardization of clinical practice for infants at the EOL. Existing infant EOL care practices include frameworks such as the American Academy of Pediatrics (AAP) clinical report, National Institute for Health and Care Excellence (NICE) pediatric EOL guidelines, and the WHO standards for newborn care, which emphasize early palliative integration, family-centered decision-making, symptom management, and bereavement support. Models such as consultative, integrative, and collaborative approaches in neonatal palliative care are widely used across academic and community systems. Common elements including advanced care planning, ethical principles, multidisciplinary coordination, and cultural sensitivity appear consistently in international guidelines, offering a strong foundation for standardizing pediatric EOL practices globally. Determination of common elements in EOL practices across healthcare systems in combination with the common elements identified in this review yields substantial promise in laying the foundation to revolutionize pediatric palliative medicine.

 This scoping review presents broad descriptive insights into current neonatal EOL practices, identification of both consistencies and inconsistencies across standards of care, and a clear demonstration that no standardized system currently exists. By examining common elements across diverse approaches, it provides a valuable foundation for future guideline development and targeted interventions.

 The primary limitation of this review is that we did not conduct a systematic review with meta-analyses. The scoping design of this review was more descriptive in nature, thereby not involving composite statistical analyses of components across sources. These limitations delimited rigorous examination of study biases across sources. It follows that we could not critically assess whether any of the guidelines, protocols and measures in the delivery of neonatal EOL care could be directly related to EOL contextual and situational outcomes for infants and their caregivers. Lastly, we reviewed studies only published in English which could certainly represent another limiting factor of this review in excluding potential articles published in different primary language. It is imperative for future reviews to employ systematic review protocols (e.g., PRISMA), incorporate meta-analytic techniques to synthesize outcomes, and apply rigorous bias and quality assessments (e.g., GRADE). Expanding searches to non-English and gray literature and linking guideline components to measurable outcomes will strengthen evidence and reduce bias.

## Conclusion

 Taking everything into consideration, there are several elements in EOL care among infants that warrant further exploration as future targets for intervention. Neonatal EOL care remains an ongoing complexity in modern healthcare, and as more infants with life-limiting illnesses navigate this vulnerable phase, the urgency to act cannot be overstated. Time is of the essence in building capacity to deliver compassionate, evidence-based care. Establishing a standardized, harmonized approach is a promising strategy, but achieving this requires global collaboration, robust pilot feasibility studies, and widespread knowledge dissemination.

 To drive meaningful change, stakeholders must be actively engaged across both inpatient and community settings. In hospitals, this includes interdisciplinary training for clinicians, integration of palliative care consults early in the care trajectory, and embedding family-centered decision-making frameworks. In community settings, partnerships with home health agencies, hospice programs, and parent advocacy groups can foster continuity of care and culturally sensitive support. Creating forums for dialogue such as regional collaboratives, virtual learning networks, and global task forces will accelerate consensus-building and guideline development.

 Continued investigation and coordinated action can pave the way for globally implemented neonatal EOL guidelines, advancing care quality and aligning with the WHO’s mission to optimize EOL care for all populations. Through shared commitment and collaboration, we can transform neonatal EOL care into a model of equity, compassion, and excellence worldwide.

## Competing Interests

 We declare that we do not have any competing interests.

## Ethical Approval

 This article does not contain any studies with human participants or animals performed by any of the authors.
